# Psychological difficulties of LVAD patients and caregivers: A follow up over one year from discharge

**DOI:** 10.1111/aor.14071

**Published:** 2021-09-29

**Authors:** Silvia Rossi Ferrario, Anna Panzeri, Massimo Pistono

**Affiliations:** ^1^ Unit of Psychology‐Neuropsychology IRCCS ICS Maugeri Institute of Veruno Veruno Italy; ^2^ Department of General Psychology University of Padua Padova Italy; ^3^ Unit of Cardiology IRCCS ICS Maugeri Institute of Veruno Veruno Italy

**Keywords:** caregivers, clinical psychology, follow‐up, LVAD patients, psychological health, rehabilitation

## Abstract

**Background:**

After the rehabilitation program, patients with left ventricular assist device (LVAD) are discharged home, but the adaption to the daily life with the implant is challenging, both with practical and psychological consequences. Literature is lacking detailed information about the quality of life of LVAD patients and caregivers after discharge to home.

**Objective:**

This study aimed at evaluating the post‐discharge outcomes of both LVAD patients and their caregivers in terms of quality of life, affectivity, and psychological health.

**Methods:**

In this observational follow‐up study, LVAD dyads discharged home from 1 year to 6 years were re‐contacted by phone and received by mail an envelope with self‐report questionnaires. Responses of 39 complete dyads of patients (mean age 68.59 ± 4.31; males: 92.31%) and their caregivers (mean age 61.59 ± 11.64; males: 17.95%) were analyzed.

**Results:**

Patients and caregivers reported the moderate levels of anxiety, depression, and caregiver strain, and Illness denial and conscious avoidance were associated between them. The couples often reported that the LVAD has impairments for their sleep and for their affective–sexual relationship. Caregivers often reported impairment in social life and self‐care.

**Discussions:**

Despite the satisfaction for the medical and territorial assistance, patients showed psychological difficulties such as anxious and depressive symptoms and caregivers tend to neglect themselves. Even after a long time from discharge to home, the psychological distress of LVAD patients and caregivers is still considerable. Structured and continuous psychological interventions are required to support their psychological health overtime after the discharge to home.

## Graphical abstract

In this study, Rossi Ferrario et al. explore the psychological difficulties of LVAD patients and their caregivers over 1 year from discharge from post‐implant rehabilitation. Their psychological distress levels are still moderate, with difficulties in sleep and in the affective and sexual areas. Psychological monitoring and support over time are recommended.

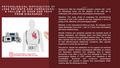

## 1 INTRODUCTION

Mechanical circulatory support devices have changed the management of acute and chronic heart failure when not improvable with medical therapy. In particular, the left ventricular assist device (LVAD) has modified the expected survival, especially in recent years.^1^ Whether they are used for the bridge to transplant (BTT) or destination therapy (DT), the results obtained in terms of life expectancy have increased, with the DT become always more frequently used.^2^

When considering an LVAD surgery, beyond the patients’ medical and neuro‐psychological condition, the presence of a designated caregiver is very important.^3^ Informal caregivers are nonprofessional unpaid figures who provide help, care, and assistance to a beloved person with an impairing illness‐related condition—such as the LVAD implant.^4^ Usually, informal caregivers are partners, family members, or close friends. LVAD caregivers are precious and sometimes necessary resources for LVAD patients in particular after discharge from the hospital to home through the post‐operative recovery time but also later on. Often caregiving continues beyond the post‐operative phase and extends up to the life‐length of the patient. Caregivers offer practical assistance with crucial everyday activities, as driveline wound care and disinfection, device management (batteries change, responding to alarms), and drug therapy administration. The caregiving intensity varies according to the patient clinical course (uncomplicate recovery vs. complications or ongoing noncardiac medical issues). Moreover, caregivers also psychologically support the patient, providing emotional reassurance and support, listening them, and simply being present together through life challenges. Interestingly, literature highlighted that patients and caregivers mutually influence each other feelings, emotions, and psychological conditions,^5^ in particular regarding the dyadic coping abilities and depression levels. Given this strong interdependence, it is important to consider both the perspectives of LVAD patients and their caregivers.^6^, ^7^ This is in line with the Dyadic Illness Management Theory^8^ proposing an inclusive model of illness‐management with a dyadic approach to understand how both members of the dyad are interconnected and can reciprocally influence their psychological and health outcomes.

Interestingly, the literature highlighted the key role of psychological and social factors in contributing to the functional adaptation process to the illness condition, both for patients and caregivers.^9^, ^10^, ^11^ Beyond the importance of psychological factors for mental health, psychological factors can also promote motivation and adherence to treatments and clinical exams,^12^ thus with substantial positive consequences for the physical health of patients with LVAD and their caregivers.^4^

Through all the process of adaptation to the LVAD, patients and caregivers have to face several challenges. Patients may experience body image alterations, they may suffer from a lack of autonomy in activities of daily living, and they can feel like a burden to caregivers. Caregivers have to sustain a multi‐faceted strain characterized by reduction of time dedicated to other activities (eg, leisure‐time, work activity, interpersonal relationships), emotional burden (eg, uncertainty, worries, sadness, loneliness).

Both patients and caregivers can experience affective and sexual difficulties, and they often have to rediscuss their personal and societal identity (eg, changes in family roles, lack of return to full‐time employment).^13^, ^14^, ^15^ All these factors associated with this illness‐related condition can generate a variety of feelings and emotions. On one hand, the negative emotions include anger, fear, denial, uncertainty, anxiety, and sadness.^14^, ^16^, ^17^, ^18^ On the other hand, some can develop positive feelings and emotions like gratitude, well‐being, and positive post‐traumatic growth. Moreover, evidence showed that some protective factors—social support, coping—are still associated with better psychological health which is in turn associated with better physical outcomes.^19^, ^20^, ^21^ All these factors contribute to a trajectory of functional or impaired adaptation that may lead to the development of severe psychological and psychiatric issues, like anxiety and depression.^10^, ^18^

According to Abshire’s review,^22^ patients with LVAD and their caregivers have to face a journey through four distinct phases. The *Pre‐LVAD phase* goes from the first discussions for the LVAD to its implantation. The *Implant Hospitalization phase* concerns the medical and rehabilitative process where the patient is (almost) fully dependent on the professional caregivers.

After discharge to home, there are two phases, literature showed that returning home requires a great effort to adapt, both in the short and long term.^22^

*Early Home Adaptation* is characterized by experimenting and developing routines for daily living activities, adapting the skills acquired in the hospital, and slowly approaching independence and autonomy. In this stage, the family caregiver is a necessary figure, both practically and emotionally. Also, post‐surgery follow‐up care encompasses distressing, sometimes unexpected, and frequent clinic visits, exams, and travels. Home privacy allows partners to re‐explore affectivity and sexual intimacy, but these aspects may be controversial due to physical and psychological difficulties (eg, body image).

In the *Late home Adaptation phase*, patients and caregivers gain growing confidence and increased autonomy in self‐management and activities of daily living, including device manipulation. Nonetheless, LVAD patients show difficulties in resuming previous individual and social roles, both in the family and the job‐related sphere—indeed, most LVAD patients do not return to work. Feelings of anxiety and sadness can arise.

In the *Late home Adaptation phase,* some patients elaborate their illness‐related condition, they adapt to a change in their sense of normalcy and can functionally elaborate their condition, reaching acceptance of their condition, up to also forgiving the situation (ie, illness)^23^, ^24^, ^25^ and even developing gratitude for the LVAD.^26^ Differently, other patients may experience difficulties in adapting to this “new life,” up to developing psychological distress, sadness, solitude, hopelessness up to suicidal thoughts and attempts.^5^, ^18^

Despite the increasing number of implanted patients and the importance of the psychological factors, most studies focused only on the first phases of *Pre‐Implant* and *Hospitalization,* while few studies focused on the phases of *early home* and *long‐term adaptation*. Some data suggest that, during the first months after the implant, patients seem to improve the self‐perceived quality of life (QOL)^20^ and to maintain this result over time together with a better emotional state.^27^ In this period, also caregivers seem to reduce their perceived strain,^20^ even if some authors point out that their psychological well‐being still results to be impaired when compared with the general population.^9^ However, these results are far than exhaustive and the psychological health and the QOL of patients and caregivers once at home is still poorly studied and thus needs to be improved.^28^

It is important to explore and understand the progression of psychological health and QOL of both LVAD patients and caregivers over time. According to previous literature,^20^, ^22^ the most relevant areas to assess over time are both medical, assistance‐related, psychological, and social. In particular, the satisfaction with the territorial health structures, the perceived cognitive efficiency, the autonomy in activities of daily living (ADL), the sleep difficulties, psychological symptoms of anxiety, depression, and denial, as well as the LVAD repercussions for affectivity and sexuality.^29^ Moreover, the recent COVID‐19 pandemic has represented a critical circumstance for people and frail patients in particular,^30^, ^31^, ^32^, ^33^, ^34^, ^35^, ^36^ including the ones with LVAD^37^, ^38^—exposed to higher complications—and who experienced a considerable reduction in routine clinical activities with the risk of laxer connections and poorer communications with the LVAD referring center.^34^, ^37^, ^38^, ^39^

Given this background, this observational study aimed to explore and describe the post‐discharge outcomes of patients with LVAD and their caregivers who were facing the early and long‐term phases of home adaptation after at least 1 year has passed since discharge from rehabilitation. A postal follow‐up survey aimed to better understand the psychological health and quality of life of LVAD patients and caregivers, this information is useful to inform structured and continuous psychological interventions after discharge to home.

## 2 METHODS

### 2.1 Participants

LVAD patients discharged by a rehabilitation center in northern Italy together with their caregivers were included in the study. As a routine practice of the implant and rehabilitation centers, due to their medical characteristics, these patients are not pre‐assigned to DT or BTT because this decision will be based on their post‐implant outcomes and adaptation. All the patients had modern types of devices (JARVIK, INCOR, HEART WARE, and HEART MATE III) that are easier to manage than the older models.

Inclusion criteria for patients were: age >18 y.o., correctly speaking Italian, being implanted with an LVAD, being discharged home for at least 1 year, not having clinical conditions (cognitive/sensorial deficits) preventing them from the assessment, and not having received a heart transplant yet. Also, the respective informal caregivers were enrolled. The study was approved by the Ethics Committee of the Maugeri Scientific Institutes (protocol N° 2379).

### 2.2 Procedure

After updating the list of patients implanted and rehabilitated, all the patients were contacted by phone and were informed about the study. The absence of cognitive deficits was assessed based on previous clinical history (proven chronic cognitive decline) and based on a telephone interview conducted by a psychologist with neuropsychological training aimed at assessing the main cognitive functions (space‐time orientation, speech, memory). Envelopes containing the paper questionnaires and informed consent were sent through the mail to those who agreed to participate, together with a pre‐stamped return envelope. Assistance calls were offered to help to fill the questionnaires if needed and a reminder phone call was done to those who did not send back the envelope.^40^

### 2.3 Measures

To assess the psychological conditions and the QOL of patients and caregivers, the following measures were used.

### 2.4 For patients and caregivers

The *Illness Denial Questionnaire* (IDQ)^41^, ^42^ is a validated instrument to evaluate denial of negative emotions (5 items, eg, “*I am angry because of this condition/illness*”), resistance to change (4 items, eg, “*Life does not change with this condition/illness*”), and conscious avoidance (6 items, eg, “*The best way to deal with this condition/illness it not to think about it*”). The first two represent the core components of illness denial while the last seems to reflect an initial step toward awareness. The response format was ‘True’ (= 1)/‘False’ (= 0). Higher scores are associated with higher levels of the measured constructs. The Cronbach’s alpha was good for all the scales (denial of negative emotions α = .76, resistance to change α = .66, and conscious avoidance α = .71).

The *Satisfaction for the local medical assistance* (SLM) in the last month was measured with 4 items (eg, “*In general, during the last month how much were you satisfied with the assistance received by your general practitioner?*”) scored on a 4‐point Likert scale from “Not at all” (= 1) to “A lot” (= 4). High scores indicate high satisfaction. The Cronbach’s alpha was acceptable (*α* = .65).

The *Activities of Daily Living* related to the management of the LVAD (ADL‐LVAD) were measured with 4 items (eg, “*In general, during the last month did you find any difficulty in the management of the LVAD*?”) scored on a 4‐point Likert scale, from “Not at all” (=1) to “A lot” (=4). Higher scores indicate higher difficulties. The Cronbach’s alpha was good (α = .70).

The *Activities of Daily Living related to the self‐care* (ADL‐SELF) included 6 items (eg, “*In general, during the last month did you find any difficulty in eating correctly and regularly*?”) scored on a 4‐point Likert scale, from “Not at all” (=1) to “A lot” (=4). Higher scores are associated with higher difficulties. The Cronbach’s alpha was good (α = .75).

The *Self‐perceived Cognitive Difficulties* (CD) were measured with items (eg, “*I* *n general, during the last month comparing with 1 month ago, do you perceive any difficulty in remembering the name of things or persons?*”), on a 4‐point Likert scale from “Not at all” (=1) to “A lot” (=4). The higher the score, the higher the difficulties. The Cronbach’s alpha was very good (α = .91).

The *Sleep Difficulties* (SD) was assessed with 3 items (eg, “*In general, during the last month how often the LVAD interfered with the quality of your sleep?*”) on a 4‐point Likert scale, from “Not at all” (=1) to “A lot” (=4). Higher scores are associated with more severe sleep difficulties. The Cronbach’s alpha was good (α = .71).

The *Affectivity and Sexual Relationships* (ASR) were measured with 5 items (eg, “*In general, during the last month did you have any difficulty in exchanging affectionate gestures (like kisses, hugs) with your partner because of the LVAD?*”; “*In general, during the last month, how much did the LVAD compromise your sexuality?*”) on a 4‐point Likert scale from “Not at all” (= 1) to “A lot” (= 4). Higher scores are associated with higher difficulties. The Cronbach’s alpha was acceptable (α = .68).

The *COVID‐19 psychological distress* (C19‐PSY) was assessed because of the unforeseen pandemic that occurred during the data collection. Two preliminary items asked if the respondent or a beloved one resulted positive to COVID‐19 with a yes/no response format, and 7 other items (eg, “*In general, are you feeling anxious because of the COVID 19*?”) assessed the psychological impact of COVID‐19 on a 4‐point Likert scale from “Not at all” (= 1) to “A lot” (= 4). Some examples of items are “*COVID‐19 makes me sad*” and “*I was afraid of my health*”. Higher scores indicate higher psychological distress. The Cronbach’s alpha was very good (α = .88).

### 2.5 Only for patients

The *Anxiety and Depression Reduced version* (AD‐R),^43^ a validated instrument to evaluate state anxiety (10 items, 4‐point Likert scale, from “Not at all” = 1 to “A lot” = 4, example: *“I feel calm”*) and depressive symptoms (15 dichotomous items, yes = 1/no = 0, examples: *“I feel sad”, “Life is worth living”*). Higher scores are associated with more severe symptoms. In particular, the discriminant clinical cut‐off for state anxiety is 22 for males and 25 for females. For depressive symptoms, the cut‐off is 7 for males and 9 for females. The α was .71 for anxiety and .86 for depression.

### 2.6 Only for caregivers

The *Family Strain Questionnaire Short Form* (FSQ‐SF),^44^ a validated instrument to evaluate the caregiver’s strain (30 dichotomous items yes = 1/no = 0). Some examples of items are: *“Nobody understand the burden I am carrying,*” “*I am worried about the patient’s illness,*” “*I would like to have more time for myself*.” Scores are distributed in four areas, with the areas SR (Strongly Recommended, from 13 to 20) and U (Urgent, from 21 to 30), indicating the need to refer the caregiver to psychological/psychiatric consultation. A higher score is then associated with higher caregiver strain. The Cronbach’s alpha was very good (α = .91).

### 2.7 Statistical analysis

Only the questionnaires completed by both the dyad members were analyzed. The descriptive statistics were performed to analyze the sociodemographic characteristics of the sample and the qualitative sections of the questionnaires (SLM, ADL‐LVAD, ADL‐SELF, CD, QS, ASR, C19‐PSY) as well as to verify the normality of data distribution. Correlations and *t*‐tests were performed to explore the relations between the measures of anxiety, depression, caregiver strain, and illness denial. The time since the implant and the impact of COVID‐19 was considered as additional information to compare the psychological reactions through a linear model.

## 3 RESULTS

Among 248 patients implanted with LVAD and rehabilitated (224 males, 90.32%), at the time of this study 29 (11.69%) received cardiac transplants and 119 (47.98%) deceased.

The remaining 100 patients were contacted by phone: some did not answer (*n* = 24), some refused (*n* = 10), and a total of 66 patients accepted to participate in the study. These 66 patients received at home an envelope containing the questionnaires. Unfortunately, 2 of them died and 3 had severe health issues (hospitalization because of falls and/or hemorrhages).

**FIGURE 1:**
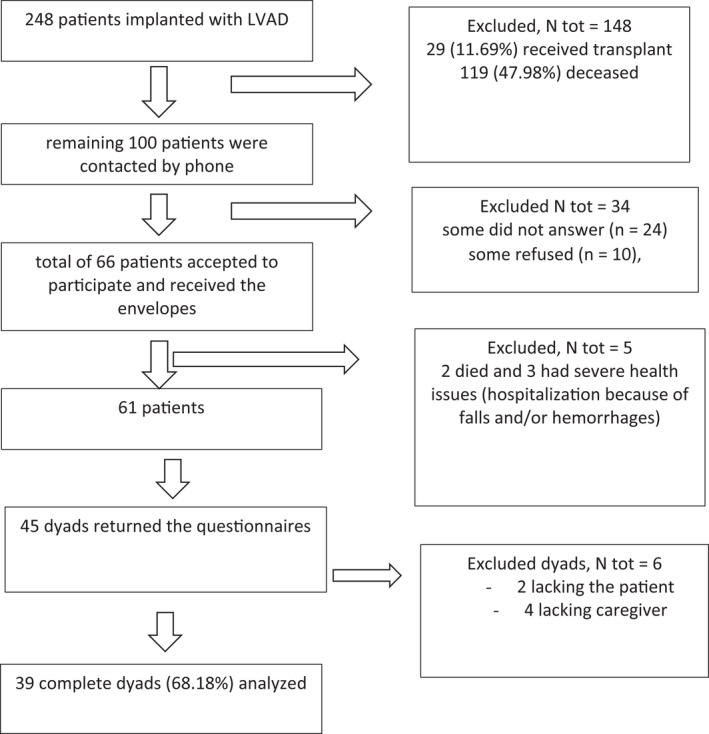
Study flow diagram

Out of 61 patients, only 45 returned the envelopes that contained the questionnaires of 39 complete dyads (68.18% of those sent). The response rate to this mail survey is in line with other similar studies.^40^, ^45^ Figure 1 shows the study flow diagram.

Then, a total of 78 subjects (39 patients and their 39 caregivers) were analyzed. Descriptive statistics revealed that the data distribution was normal. Table 1 gives the characteristics of the analyzed sample—it did not differ in age and sex from the patients not willing to participate (Table S1). Patients were prevalently males, older than caregivers (*p *< .001), and implanted since 3.45 ± 1.67 years, (range 1–6). Half of them (*n* = 18/39; 50%) reported nonsurgical adverse events following the implant, such as cardiac complication (*n* = 10/39; 25.64%), infections (*n* = 6/39; 15.38%), and stroke (*n* = 3/39; 7.69%). This type and rate of medical and physical complications is in line with what LVAD patients and their caregiver usually experience, these experiences may impact on the patients’ and caregivers’ psycho‐physical health.

**TABLE 1:** Characteristics of the sample

Variable	Patients (*n* = 39)		Caregivers (*n* = 39)	
Age, mean (SD)	68.59	4.31	61.59	11.64
*Gender*, *n* (%)				
Male	36	92.31%	7	17.95%
Female	3	7.69%	32	82.05%
*Marital status*, *n* (%)				
Single	1	2.56%	6	15.38%
Married	33	84.61%	33	84.62%
Widow	5	12.82%	0	0
*Education level*, *n* (%)				
Elementary	7	17.95%	5	12.82%
Middle school	14	35.90%	13	33.33%
High school	13	33.33%	20	51.28%
Degree	5	12.82%	1	2.56%
*Employment*, *n* (%)				
Working	0	0	7	17.95%
Retirement	36	92.31%	22	56.41%
National economic assistance	14	35.90%	–	–

Fourteen patients (35.90%) received economic help—as a disability allowance—from the government.

Caregivers were mostly females (*n* = 32, 82.05%), prevalently the patients’ spouses (*n* = 34, 84.62%), and retired (*n* = 22, 56.41).

Considering the psychological measures, patients’ state anxiety and depression showed high values, above the clinical cut‐off for the general population (Table 2). Older patients displayed worse symptoms of depression (*r* = 0.34, *p* = .032). Furthermore, the scores of anxiety and depression were positively and significantly associated with the caregivers’ strain, indicating that when the patient has emotional difficulties, also caregivers show higher distress levels (Table 3). In particular, it is important to underline that caregivers maintain a high level of distress over time, as measures with the FSQ‐SF scale (Table 2). In fact, they prevalently remain in the SR/U areas, suggesting the strong need for psychological/psychiatric counseling.

**TABLE 2:** Psychological measures

Variable (range)	Patients		Caregivers		Correlation	*t* test
	Mean	SD	Mean	SD		
*Anxiety* (10–40)	21.46	5.614	–	–	–	–
Below cut‐off, *n* (%)	19	48.7%	–	–	–	–
Above cut‐off, *n* (%)	20	51.3%	–	–	–	–
*Depression* (0–15)	5.564	3.582	–	–	–	–
Below cut‐off, *n* (%)	24	61.5%	–	–	–	–
Above cut‐off, *n* (%)	15	38.5%	–	–	–	–
*FSQ‐SF* (1–30)	–	–	13.64	8.57	–	–
Urgent	–	–	8	20.5	–	–
Strongly recommended	–	–	13	33.3	–	–
Recommended	–	–	10	25.6	–	–
Ok	–	–	8	20.5	–	–
*IDQ:* *Denial of negative emotions* (0–5)	2.153	1.694	1.769	1.422	*r* = 0.539	*t* = 1.085
					*p* < .001	*p* = .281
*IDQ:* *Resistance to change* (0–4)	0.897	1.095	1	1.256	*r* = 0.363	*t* = −0.384
					*p* = .023	*p* = .702
*IDQ:* *Conscious avoidance* (0–6)	3.72	1.85	4.08	1.99	*r* = .478	*t* = −0.825
					*p* = .002	*p* = .412

Note: the last two columns titled ‘Correlation’ and ‘t test’ refer to the comparison between the values of patients and their caregivers. Abbreviation: FSQ‐SF, Family Strain Questionnaire Short Form; IDQ, Illness denial questionnaire.

**TABLE 3:** Psychological impact of COVID‐19

COVID‐19 distress (7–28)	Patients		Caregivers	
	Mean	SD	Mean	SD
I was afraid for my health	2.69	0.95	2.79	0.92
I was afraid for the health of my loved ones	3.26	0.72	3.31	0.69
I’ve been through a bad lockdown	2.10	1.09	2.03	0.93
COVID‐19 makes me anxious	2.21	1.00	2.33	0.81
COVID‐19 makes me feel sad	1.89	0.98	2.18	0.79
I’m afraid of COVID‐19	2.62	1.04	2.72	0.88
I think there will be a second wave	3.08	0.90	3	0.81
Total	15.18	4.37	18.53	3.75

Note

None of the participants tested positive to COVID‐19.

As expected, patients’ state anxiety and depression, as well as caregivers’ strain, were negatively related with Denial of Negative Emotions and Resistance to Change, representing the core components of illness denial (Table 3).

Table 4 presents the associations between the psychological variables. Both patients and caregivers as dyads showed the same defense pattern: in fact, they share comparable levels of Denial of Negative Emotions (DNE), Resistance to Change (RC), and Conscious Avoidance (CA). Noteworthy, caregivers show significantly less strain as the patients’ core components denial (DNE, RC) increase. Also, time since the LVAD implant was not significantly correlated with any of the other measures (*p* < .05). Table 5 presents the data about the qualitative section of the assessment.

**TABLE 4:** Pearson’s Correlations among patients and caregivers

			**Patient**					**Caregiver**			
					**IDQ**				**IDQ**		
			**Anx**	**Dep**	**DNE**	**RC**	**CA**	**FSQ**	**DNE**	**RC**	**CA**
**Patient**		**Anx**	–	0.70***	−0.69***	−0.58***	−0.14	0.44**	−0.37**	−0.26	0.14
		**Dep**	0.70***	–	−0.74***	−0.55***	−0.35*	0.44**	−0.37**	−0.40*	0.05
	**IDQ**	**DNE**	−0.69***	−0.74***	–	0.63***	0.33*	−0.38*	0.54***	0.37*	0.05
		**RC**	−0.58***	−0.55***	0.63***	–	0.17	−0.33*	0.46**	0.36*	−0.08
		**CA**	−0.14	−0.35*	0.33*	0.17	–	−0.31	0.19	0.16	0.48**
**Caregiver**		**FSQ**	0.44**	0.44**	−0.38*	−0.33*	−0.31	–	−0.66***	−0.65***	−0.06
	**IDQ**	**DNE**	−0.37**	−0.37**	0.54***	0.46**	0.19	−0.66	–	0.74***	0.10
		**RC**	−0.26	−0.40*	0.37*	0.36*	0.16	−0.65***	0.74***	–	0.06
		**CA**	0.14	0.05	0.05	−0.08	0.48**	−0.06	0.10	0.06	

Note: the yellow color shows the correlations of the measures within the patient’s sample. The light blue color shows the correlation within the caregivers’ sample. The green color shows the correlations between the patients’ sample and their caregivers sample.

Abbreviations: Anx, state anxiety; CA, conscious avoidance; Dep, depressive symptoms; DNE, denial of negative emotions; FSQ, Family Strain Questionnaire Short Form; IDQ, Illness Denial Questionnaire; RC, resistance to change.

* *p* < .05

** *p* <. 01

*** *p* < .001.

**TABLE 5:** Qualitative measures

Variable (range)	Patients			Caregivers		
	M	SD	NA	M	SD	NA
**Satisfaction of territorial assistance**	**In the last month, overall, how satisfied you are with the assistance provided by:**					
Your general practitioner	3.27	0.90	2	3.25	0.87	3
Your referral hospital	3.63	0.60	4	3.61	0.77	3
Your ASL	2.83	0.87	9	2.78	1.09	12
Home nursing care	3.41	1.02	10	3.27	1.18	6
**ADL LVAD**	**In the last month, overall, you have found it difficult to:**					
Managing the VAD by my self	1.46	0.80	2	1.17	0.45	3
Managing the patient drug therapy	1.35	0.79	2	1.16	0.44	2
Contact the reference hospital	1.16	0.38	1	1.18	0.56	1
Contact the engineers	1.24	0.56	2	1.21	0.71	7
**ADL self**	**In the last month, overall, you have found it difficult to:**					
Resume relations with friends	1.54	0.85	4	2.79	0.80	0
Take back the old hobbies	2.41	1.07	3	2.39	0.91	1
Engage in new hobbies	2.48	1.12	6	1.60	0.88	1
Managing my drug therapy	–	–	–	3.14	0.91	4
Exercise regularly	2.45	1.08	1	2.37	0.94	1
Eating properly	1.51	0.76	2	3.03	0.63	0
Do my medical checks	1.57	0.96	2	2.90	1.10	0
Driving the car	1.44	0.89	12	–	–	–
**Sleep difficulties (1–12)**	**How often in the last 4 weeks did…**					
You had trouble falling asleep	1.76	1.17	1	1.79	0.98	–
You wake up frequently at night and had trouble getting back to sleep?	2.18	1.06	1	2.08	1.06	2
The VAD interfere with your sleep quality?	1.36	0.81	1	1.71	1.00	5
**Affectivity sex (0–8)**	**How often in the last 4 weeks did…**					
You have trouble exchanging affectionate gestures (hugs, kisses, effusions) with your partner because of VAD?	1.51	1.09	4	1.61	1.02	8
You experienced sexual desire/interest?	2.26	1.12	8	1.77	1.15	19
You have difficulties in having sex?	2.86	1.39	17	2.38	1.61	18
You felt uncomfortable about your sex life?	2.85	1.43	13	1.95	1.52	18
The VAD compromised your sexuality?	3.07	1.33	11	2.80	1.58	16
**Cognitive difficulties (4–15)**	**Compared with a month ago:**					
Do you feel like you’re having memory difficulties?	1.69	0.69	0	1.59	0.82	0
Do you feel like you’re having difficulty in concentration?	1.69	0.73	0	1.59	0.72	0
You seem to have trouble remembering names of things or people?	1.79	0.83	0	1.51	0.79	0
Is it hard to remember the date?	1.87	0.92	0	1.33	0.66	0

Abbreviation: NA, not applicable.

Satisfaction about the territorial assistance received was expressed by patients and caregivers, who also reported minimal difficulties in the management of the LVAD.

As often reported in the caregiving literature^46^, ^47^ caregivers reported limitations in resuming relationships with friends, bad nutrition, and poor adherence to their drug therapy and medical controls.

Sleep seemed to be disturbed for frequent awakenings both for patients and caregivers, who also stated that the device caused sleep difficulties.

Regarding the affective and sexual relationship, almost half of the sample avoided answering, indicating that this is a still delicate topic. Observing the obtained answers, it seemed to emerge that, despite a good affective relationship, the device interferes with patients’ and caregivers’ sexuality, compromising intimacy and comfort, for patients in particular.

No relevant perceived cognitive difficulties were reported by patients and caregivers.

Concerning the C19‐PSY, no subject or their relatives were affected by the virus. Exploring the influence of these answers on the psychological measure (AD‐R, FSQ‐SF, IDQ), no effect was found.

## 4 DISCUSSIONS

This study aimed at exploring the QOL of rehabilitated LVAD patients and their caregivers, from a multidimensional point of view, once at home for at least 1 year.

The results suggest that both patients and caregivers still show moderate levels of emotional distress over time—as in line with literature^14^—and that they help themselves and each other throughout the mechanism of denial. Denial is a defense mechanism that allows to protect a person from the negative emotions triggered by something that he/she is still not ready to face (eg, illness, pain, limitations). Denial might be adaptive if used for a short time, but it can become dysfunctional if it is prolonged for a long time. Indeed, denial can interfere with medical compliance (skipping medical checks) and adherence to therapy (not taking pills), thus leading to worse physical and psychological outcomes. A previous study^16^ showed that denial is common among LVAD patients and caregivers during rehabilitation, but the present study is the first one that highlights the presence of a denial mechanism also after more than 1‐year post‐discharge up to 6. These findings suggest that the process of acceptance of illness is long and complicated and these patients and caregivers need to be monitored and supported over time to favor the adaptation to their condition.

The psychological distress related to the current pandemic condition^48^, ^49^ did not significantly affect the results.

Considering the items descriptives in Table 5, it is noteworthy that the sample analyzed reported satisfaction for territorial assistance received and minimal difficulties in the management of the device. This may suggest that even when patients and caregivers are satisfied with medical attention, do not experience a sense of abandonment, or a sense of insecurity due to the device, they still can display an alarming psychological condition with moderate distress levels across different areas.

Concerning the differences between patients and caregivers, they showed similar items statistics (Table 5) but with some slight differences in some items.

The patients reported more difficulties in engaging in new hobbies than caregivers. Patients with LVAD sometimes lead a withdrawn and cautious life, instead, they should be encouraged to experiment and be open to new things, taking charge of their lives, remembering that LVAD was implanted to allow them to (continue to) live a full and dignified life.

Patients also reported higher values of cognitive difficulties (remembering names and the date) than caregivers, but this may be a plausible effect of their higher age and of the LVAD itself.

Regarding the answers about sleeping and affection, it is evident that both patients and caregivers are quite impaired in these important dimensions of QOL because of the device. In particular, patients reported higher difficulties in the sexual area (uncomfortable, compromission) when compared with caregivers. This may reflect a gender difference (higher sexual desire in male than in women) or could be biased by the fact that half of the subjects avoided the questions about sexual behavior and affection—it is not clear if this is for embarrassment or because they stopped to live this part of life. These findings are in line with current literature^50^, ^51^ showing a considerable decrease in the level of satisfaction with sexual life after LVAD implant, and most of the patients avoid this issue with doctors. Future research about the areas of sexuality and affection with the LVAD is needed and psychological support for these issues is recommended.

Finally, what emerged about the caregivers in terms of limitation in social life (difficulties in resuming friendships) and self‐carelessness (not eating properly, not doing medical checks), is a further confirmation of what lived in general by the majority of caregivers of chronic patients who tend to neglect themselves.^9^, ^14^, ^21^

In this study, older patients showed higher depression levels which may seem in contrast with previous literature showing lower device acceptance in younger patients.^11^, ^14^ However, it should be noted that in this study the general LVAD patients’ average age (68.59 ± 4.31) was older than previous studies. At the same time, literature about elder patients with cardiovascular diseases already showed the specificity of this particular population characterized by frailty conditions and not trivial gender differences both in the psychological (eg, depression) and physical outcomes (eg, survival).^52^, ^53^, ^54^, ^55^

Importantly, in this regard, these study findings may disclose an interesting possible nonlinear relationship between age and psychological adaptation throughout the life‐cycle: younger patients may have lower psychological adaptation abilities, middle‐age patients would be more flexible and prone to adaptation, and older patients may also display difficulties in finding adaptive ways to cope with illness thus developing higher depressive symptoms.^24^, ^56^, ^57^

The principal limitations of this study consist in its observational nature with a one‐time point measurement, longitudinal studies following patients changes over time are needed.^22^, ^57^, ^58^ Moreover, the sample consisted mostly of males with a mean age that is higher than other samples in literature, thus these findings should be tested also in other samples. The high proportion of males in the sample reflects the higher prevalence of male LVAD patients in the general population but may affect the generalization of results also to the female patients with LVAD. Then, the choice to study the dyads also reduced the number of subjects considered and excluded patients who had no caregiver to involve or vice‐versa (*n* = 2 dyads). Also, no formalized tests were used to screen for cognitive difficulties, but a clinical interview conducted by phone was chosen as the most feasible at distance assessment.

Despite these limitations, this study still provided an interesting perspective about the psychological health of LVAD patients and caregivers in the long‐term post‐discharge life. Both of them showed moderate distress levels. These findings highlight the need to develop strategies to reduce the risk of psychological distress in LVAD patients and caregivers.

Some strategies are recommended to prevent and mitigate the development of psychopathological symptoms. Before the LVAD implant, they should have a strong treatment motivation, adequate social support, and a strong preoperative education about life after the LVAD implant. Also, patients and caregivers should be pre‐instructed about actively seeking psychological help in the future, if needed.^59^ Indeed, a consistent number of patients and caregivers expressed severe distress when contacted by phone and were encouraged to seek professional help for their mental health despite the possible associated fears and/or stigma.^60^, ^61^, ^62^, ^63^

Also after LVAD implantation is important to monitor and assess the potential satisfaction or regret about LVAD to detect early signs of psychological distress identify those individuals requiring psychological support—with particular attention for the destination therapy patients.^18^

Future research may benefit from short and accurate assessment tools^64^, ^65^ to measure and monitor over time the several variables implied the process of adaption to illness, both the psychological (eg, depression, uncertainty, hopelessness, emotion regulation) and somatic ones (eg, fatigue, frailty).^66^, ^67^, ^68^ In particular, uncertainty in illness characterizes the experience of patients and caregivers facing illness conditions—cardiac, oncological, neurological—because of the intrinsic uncertainty about the prognosis and the future.^66^, ^69^

## 5 CONCLUSIONS

Despite the LVAD allowed achieving great physical improvements (eg, expected survival) for patients with severe heart failure, to date the research attention should be focused on the psychological health of these patients and their caregivers as well. Indeed, it is important to deepen the daily quality of life of both patients and caregivers over time, including aspects that are a fundamental part of everyone’s life, as sleeping, affection, and sexuality. Regarding the psychological measures, denial mechanisms seem to play an important role in the adaptation process and need to be considered and furtherly addressed by future research and clinical practice as well.

Finally, this study’s findings are in line with the increasing amount of international literature about caregiving that for many years has advised about the caregivers’ impairment in social life and self‐care, once again highlighting that structured psycho–social interventions should be set up and included in the routine territorial care to meet the caregivers’ needs and improve their psychological health.

## CONFLICT OF INTEREST

Authors have no conflict of interest to disclose.

## AUTHORS CONTRIBUTIONS

SRF and AP conceived the study, AP and MP did the data collection, AP and SRF did data analysis and interpretation, AP and SRF drafted the article. All the Authors provided critical revision and approved the article.

## Supporting information

Table S1
