# Supplementary material for: Psychological difficulties of LVAD patients and caregivers: A follow up over one year from discharge
Source: Artif Organs. 2021 Sep 29;46(3):479–90. doi: 10.1111/aor.14071 (PMC9292387; doi:10.1111/aor.14071)
Supplement: Supplementary file 1 — Table S1 [file AOR-46-479-s001.docx]

**Supplementary materials**

**Table 1S. Comparison of the included and excluded patients.**

|  |  | **Excluded**  **(n 27)** |  | **Included**  **(N 39)** | ***p* value** |
| --- | --- | --- | --- | --- | --- |
| **Age mean (sd)** |  | 69.11 (6.64) |  | 68.28 (5.07) | > 0.05 |
| **Min-max** |  | 56-79 |  | 53-78 | - |
| ***Sex*** |  |  |  |  | > 0.05 |
| **Male** |  | 23 (85.19%) |  | 36 (92.31%) |  |
| **Female** |  | 4 (14.81%) |  | 3 (7.69%) |  |

**Note:** the reported p-values is calculated with independent t test for age and with chi-square with Montecarlo correction for sex.
